# On the Various Risks of Operations

**Published:** 1868-02

**Authors:** James Paget


### ARTICLE VII.

### *On the Various Risks of Operations.*

By James Paget, F. R. S., (delivered at St. Bartholomew's Hospital.)

Lect. I. Part II.—The foregoing, so far as I have been able to learn them, are the various risks of patients with admitted morbid constitutions. The importance of being able to decide the questions arising in such cases must be evident to you. And questions of equal importance, and yet of greater difficulty, arise in the cases of many who may not be called diseased, but who certainly are not in any just sense of the word healthy, such as the plethoric, the over-fat, the intemperate, the over-fed, the feeble, the degenerate, the cold-blooded. What can be safely said about these, and of the dangers they severally incur when we wound them? I will try to tell what I believe.

Plethora, pure and simple, is not a bad condition for operations. So far as I have seen, people that have been full-blooded, ruddy, warm, round-limbed, tight-skinned, with strong hearts, and, as we suppose, a rather excess of blood, have done well. But such people must be carefully managed ; not fed too well ; not kept too long in bed ; not allowed to retain their refuse ; and mere bigness must not be taken for plethora.

For the over-fat are certainly a bad class, especially when their fatness is not hereditary, but may be referred in any degree to their over-eating, soaking, indolence, and defective excretions. The worst of this class are such as have soft, loose, flabby, and yellow fat ; and I think you may know them by their bellies being pendulous and more prominent than even their thick, subcutaneous fat accounts for ; for this shape tells of thick omental fat ; and, I suppose, of defective portal circulation. I know no operations in which I more nearly despair of doing good than in those for umbilical hernia or for compound fractures in people that are over-fat after this fashion. Nothing short or the clearest evidence of necessity or of great probable good, should lead you to advise cutting operations in people of this kind. Do lithotrity for them rather than lithotomy ; incline against amputations for even bad compound fractures ; and, wherever you can—as, for instance, cutaneous cysts, hæmorrhoids, and the smaller examples of scirrhous mammary cancers—use caustics rather than the knife or ligature.

All these warnings must be doubled for the intemperate. One does, indeed, sometimes meet with habitual drunkards who pass safely through the perils of great operations ; but these are rare exceptions to the rule, according to which one may reckon that the risks of all operations increase with the increasing degrees of habitual intemperance. I think you will find that a habit of slight intemperance is much worse than occasional great excesses ; that regular soaking is worse than irregular carousing; probably because of the steady impairment of the blood and of all the textures to which the soaking leads. Of course you will keep your hands off notorious drunkards, unless you are driven by the stress of a stangulated hernia, or stopped windpipe, or something leaving you as little choice as these do. But you must be on your guard to detect a good deal of drunkenness of the soaking kind which is not notorious and not confessed. Be rather afraid of operating on those, of whatever class, who think they need stimulants before they work; who cannot dine till after wine or bitters; who always have sherry on the sideboard; or are always sipping brandy-and-water; or are rather proud that, because they can eat so little, they must often take some wine. Many people who pass for highly respectable, and who mean no harm, are thus daily damaging their health, and making themselves unfit to bear any of the storms of life.

On all such as these, operations are more than doubly hazardous. Of course you may hear of wondrous escapes from dangers, and, on the credit of a few exceptions, silly proverbs are made about the impunity of drunkards; but the general rule is certain. Every risk of an operation is increased in the habitually intemperate; they are, above the average, liable to every one of all the sources of danger and of death.

I have had no sufficient experience among teetotalers to enable me to speak with any certainty of their capacity for bearing operations. I cannot doubt that a patient trained all his life, to habits of rigid temperance would bear injuries of all kinds better than the average of men; but people of this sort are not commonly those with whom you have to do under the name of teetotalers. These are, much more commonly, such as have been intemperate, or, to say the least, imprudent in their manner of living, and have then wholly changed their habits, and lived without any stimulants whatever. Of such people I have no good opinion when they come to be the subjects of surgery; for they seem to retain the bad liabilities of the intemperate long after they have given up their bad habits. I would not adopt the opinion that I have heard some express, that teetotalers are worse patients than drunkards; but I should always expect that a very long period of reformation would be required to free a man from the damages he has sustained by intemperance.

Over-eating is not commonly supposed to lead to any such risks of life as over-drinking does; yet I believe that you will find, in operative surgery, that among the habits that increase the risks of life, this may stand not far off drunkenness, especially if the other-eating is of meat and other nitrogenous foods. I am led to believe this from several cases that I have observed, and I think that there are large evidences of it. You know that the general results of operations in provincial hospitals tell of a smaller mortality than in the hospitals of London and the largest towns. The difference is commonly ascribed to differences in the purity of the air, and other advantages of that kind in the comparatively rural districts. I believe that much more of it is due to the differences of habits in the several classes of patients. The differences are many; but one of the chief of them is that of the poor in the agricultural districts eat far less meat than those in large towns do, and are, by comparison, less fed, though probably not worse fed, and you may frequently observe that patients who come to us from agricultural districts bear operations in all respects, better than Londoners who are submitted to the same proceedings. Of course many things occur to make the differences of constitution between a town and a country population; but I am satisfied that among these things a very potent influence is exercised by the difference of diet. And the differences that we may thus see are strongly illustrated by what one hears of the results of operations upon the natives of India and other Eastern countries, whose diet is almost exclusively vegetable. Almost any amount of injury may be inflicted on them, and not be followed by the destructive mischiefs which occur in Europeans under the same circumstances. They are defective, it is said, in healing power; but they recover with comparative certainty, however slowly, from operations of the greatest magnitude. A common expression about them is, "You can't kill them."

(To be Continued.)

